# Structure-Guided Engineering of Protein VP2 from Epizootic Hemorrhagic Disease Virus Maximizes Production and Confers Complete Protection as Subunit Vaccine

**DOI:** 10.3390/vaccines14010007

**Published:** 2025-12-20

**Authors:** Samuel Jurado, Luis Jiménez-Cabello, María del Carmen Nuñez, Sergio Utrilla-Trigo, Eva Calvo-Pinilla, Iván Mazuecos-Aragonés, José Ramón Gutierrez, Ana Falcón, Javier Ortego, José M. Escribano

**Affiliations:** 1 Alternative Gene Expression S.L. (ALGENEX), Ronda de Poniente 14, 28760 Madrid, Spain; samuel.jurado@algenex.com (S.J.); carmen.nunez@algenex.com (M.d.C.N.); joseramon.gutierrez@algenex.com (J.R.G.);; 2 Centro de Investigación en Sanidad Animal (CISA), Instituto Nacional de Investigación y Tecnología Agraria y Alimentaria (INIA), Valdeolmos, 28130 Madrid, Spain; lfj.cabello@inia.csic.es (L.J.-C.); sergio.utrilla@inia.csic.es (S.U.-T.); calvo.eva@inia.csic.es (E.C.-P.); ivan.mazuecos@inia.csic.es (I.M.-A.); ortego@inia.csic.es (J.O.)

**Keywords:** Epizootic hemorrhagic disease virus, protein VP2, baculovirus vector, *Trichoplusia ni*, insect pupae, subunit vaccine

## Abstract

Epizootic hemorrhagic disease (EHD) is an important livestock disease caused by Epizootic hemorrhagic disease virus (EHDV). The recent incursion and wide distribution of EHDV in Europe have increased the need for effective vaccine candidates. **Background/Objectives:** The VP2 protein of EHDV forms the outer capsid layer of the virion and is essential for viral assembly and host cell entry. Owing to its antigenic properties, VP2 represents a major target for vaccine development. However, the recombinant production of VP2 is limited by low stability and poor yields, representing a significant barrier for the generation of safe and effective subunit vaccines. **Methods:** To overcome these limitations, the VP2 protein from EHDV serotype 8 (EHDV-8) was rationally engineered with targeted modifications at both the amino and carboxyl termini of its coding sequence. Recombinant expression was performed using a baculovirus vector-mediated system in *Trichoplusia ni* pupae (CrisBio^®^ technology), employed as living biofactories. **Results:** The engineering of VP2 resulted in up to a tenfold increase in protein yields compared with the wild-type sequence, while maintaining the trimeric structural integrity of the recombinant protein. Both wild-type and engineered VP2 protein variants were formulated and used to immunize IFNAR(−/−) mice, a model susceptible to EHDV infection. Both engineered and wild-type VP2 formulations elicited comparable neutralizing antibody responses in vaccinated animals. Furthermore, immunization with either formulation conferred full protection against lethal EHDV-8 challenge. **Conclusions:** In this work, we demonstrated that the rational engineering of the VP2 protein significantly improved recombinant expression yields in a baculovirus-based system without compromising structural integrity or immunogenicity. These findings additionally demonstrate the feasibility of producing high-quality VP2 antigens in *T. ni* pupae using CrisBio^®^ technology and support their potential application in the development of subunit vaccines against EHDV.

## 1. Introduction

Epizootic hemorrhagic disease virus (EHDV) is an emerging and severe livestock pathogen and the causative agent of Epizootic hemorrhagic disease (EHD), which is included in both the USA’s National List of Reportable Animal Diseases and the notifiable disease list of the World Organization for Animal Health (WOAH) [1,2]. EHDV affects wild and domestic ruminants. Among them, the white-tailed deer (WTD) is the most susceptible species, showing high mortality rates [3,4]. Other wild and domestic ruminants, such as cattle, also develop clinical disease following infection with EHDV [5,6,7,8,9,10]. Indeed, recent EHDV outbreaks are characterized by an increased pathogenicity in cattle populations [11]. Cattle infected with EHDV develop clinical signs such as hypersalivation, reddening and ulcers, protruded edematous tongue, coronary band injury, conjunctivitis, dyspnea, dysphagia, or recumbency, among others [12]. Globally, the economic impact caused by EHDV is significant and includes both direct economic losses, such as livestock weight loss, reduced fertility rates, significant reductions in meat and milk production, and the death of affected animals, as well as indirect economic losses due to trade restrictions [13,14]. EHDV has been isolated in North and South America, Africa, Asia, the Middle East, and Oceania, and it is considered endemic in parts of North America and Australia and in several regions of Asia and Africa [10]. Before 2000, there were almost no reports of EHDV in the Mediterranean basin [10,15]. Europe remained free of EHDV until October 2022, when EHDV-8 was reported for the first time in Sicily, southwestern Sardinia, and Spain [9,16,17,18]. Subsequently, EHDV propagated to Portugal and France [19,20]. Probably, the origin of the EHDV-8 cases in Spain and Italy came from North Africa, where the same serotype had already been reported [9,21,22]. The spread of the virus into Europe is most likely due to the introduction of infected arthropod vectors through air masses originating from Africa [23].

Like members of the *Orbivirus* genus, EHDV non-enveloped virions present a structure characterized by an icosahedral capsid, which is divided into three consecutive protein layers: the inner and intermediate layers (core) and an outer capsid [24,25]. The genome is located inside the core particle and comprises ten linear double-stranded RNA segments that encode for seven structural (VP1–VP7) and at least four non-structural proteins (NS1, NS2, NS3/NS3A, NS4, and probably the putative NS5, as is the case for BTV) [24,26,27,28]. As for the prototype BTV, the outer protein layer is made of 60 trimers of VP2, the most exposed virion protein, and 120 trimers of VP5 [29,30]. The inner capsid is made of the subcore, formed by VP3, and the intermediate layer, constituted by VP7, along with three minor structural proteins with enzymatic activities: VP1 (RNA-dependent RNA polymerase), VP4 (capping enzyme), and VP6 (helicase) [25,31,32,33,34]. The outer capsid proteins VP2 and VP5, encoded by segments 2 and 6, respectively, are the most variable proteins among EHDV serotypes, especially VP2, probably due to the great selective pressure [35]. VP2 is a key structural protein of viruses in the *Orbivirus* genus, being found in viruses such as EHDV and BTV, where it plays a critical role in viral assembly and host cell entry [36]. VP2 is responsible for receptor binding, hemagglutination activity, and the induction of neutralizing antibodies, making it the determinant of serotype specificity and a crucial target for serological diagnostics and vaccine development [37,38,39,40,41]. To date, at least seven different serotypes of EHDV have been identified, designated as 1–2 and 4–8, based on cross-neutralization assays and supported by extensive phylogenetic studies [10,35].

EHDV causes a significant economic impact in affected regions [13]. Given the potential socioeconomic repercussions of EHDV in Europe [14], it is imperative to establish a series of measures to control the transmission and spread of the virus. Among all possible control measures, once the disease is introduced in Europe, the implementation of vaccination campaigns against EHDV would be recommended. In Japan (where high prevalence of EHDV can occur, especially in cattle populations [42,43]), two vaccines have been developed and commercialized: one monovalent attenuated vaccine against EHDV-2 and another bivalent inactivated vaccine against EHDV-2 and bovine ephemeral fever virus [10,44]. In the United States, where EHDV-1 and -6 are endemic, autogenous inactivated vaccines are often used for disease control in WTD breeders, although there is no data on their effectiveness [45]. Novel vaccine approaches such as subunit vaccines should address the inherent drawbacks of classic vaccine approaches, especially those related to safety and implementation of DIVA (differentiating between vaccinated and naturally infected animals) strategies. Currently, the efficacy of a subunit vaccine against EHDV-2 capsid proteins is being studied. Preliminary results in WTD and cattle are promising, with very wide safety margins [46]. Similarly, a recombinant EHDV-8 VP2 subunit vaccine has been licensed, and recent work has shown the protective efficacy of a baculovirus-expressed recombinant EHDV-8 VP2 protein against the homologous serotype in cattle [47]. Researchers from the United Kingdom have developed subunit vaccine candidates based on EHDV-like particles (VLPs) of different serotypes, capable of inducing neutralizing antibody responses in rabbits [48,49]. The progress of EHDV through Europe implies the need for a safe, DIVA vaccine that would be effective against multiple serotypes. In this sense, a recent viral vector-based approach has highlighted the role of protein VP7 of EHDV as a potential antigen capable of inducing multiserotype protective responses against EHDV in IFNAR(−/−) mice, although it should be further confirmed in natural hosts of the disease [50]. Considering that EHDV-8 is the unique serotype currently circulating in Europe, the development of a commercial subunit vaccine based on a single protein such as VP2, combined with a diagnostic method based on other structural immunogenic proteins of the virus like VP7, would be an effective strategy to counteract the virus.

The VP2 attachment trimers of BTV adopt a triskelion shape, consisting of three tip domains radiating from a central hub domain [38,51,52]. This outer-coat triskelion is formed by three VP2 monomers, each contributing both a hub and a tip domain. Structural studies using cryo-electron microscopy have identified domains in the “triskelion leg” of VP2, where receptor-binding tip domains are exposed, including an internal sialic acid-binding pocket that facilitates host adhesion [37,38]. Structurally, the tip domain extends upward from the virion surface, while its base rests on VP7 trimers [38]. In the present study, we used the available structural information of BTV VP2 protein to design a VP2 construct from the related EHDV serotype 8, aiming to facilitate its expression and purification, with the ultimate goal of developing a subunit DIVA vaccine. Furthermore, we evaluated the immunogenicity and protective capacity of our VP2 construct against EHDV. In addition, we explore the feasibility of manufacturing the newly designed VP2 protein in *Trichoplusia ni* pupae as living biofactories (CrisBio^®^ technology, Algenex SL, Tres Cantos, Spain) [50] to develop a cost-efficient subunit vaccine based on this protein with its immunogenic features preserved.

## 2. Materials and Methods

### 2.1. Cell Lines and Viruses

Regulatory Sf9-RVN Glycobac insect cells (SIGMA-ALDRICH, St. Louis, MO, USA) were grown in ESF-AF insect medium (Expression System, Davis, CA, USA) for baculovirus generation, propagation, and titration.

Green monkey kidney cells (Vero) (ATCC, Manassas, VA, USA, Cat. No. CCL-81) and BHK-21 cells (ATCC; catalog no. CCL-10) were grown in Dulbecco’s Modified Eagle’s medium (DMEM) (Biowest, Nuaillé, France), supplemented with 2 mM glutamine (Gibco, Waltham, MA, USA) and 5% FCS (Gibco, Waltham, MA, USA).

EHDV serotype 6 (EHDV-6) (EHDV-6 MOR2006/07) and EHDV serotype 8 (EHDV-8/Spa/And/LCV-01-2022) (isolated in Spain, 2022) were used in the experiments. EHDV-8 Spanish isolate was isolated from cattle blood in KC insect cells [53] and passaged twice in BHK cells. EHDV-6 was passaged once in KC insect cells. BHK cells were used to grow virus working stocks, and virus titration and stock generation were performed as previously described [54].

### 2.2. Baculovirus Generation

The 3D structures of VP2 monomers from both BTV (serotype 3) and EHDV were generated from their complete amino acid sequences using AlphaFold2. The dissection of the structural domains of complete VP2, performed as previously described, in combination with 3D visualization, enabled the identification of cleavage points in the monomer at regions displaying unfolded secondary structure, while also allowing the deletion of entire domains such as the hub and hairpin domains. The encoding sequences for the VP2 wild-type protein and its modified version described in the present work (VP2-EHDV-8 and VP2t-EHDV-8 Mod) were synthesized by the company GenScript. The codon usage of the VP2 encoding genes was optimized for their expression in insect cells (OptimumGene™-Codon Optimization algorithm). Both sequences contained adequate flanking regions to facilitate their cloning in the pFastBac1 donor plasmid. Once the donor plasmids with the complete VP2 or modified VP2 genes were obtained, the bacmids for the generation of the different baculoviruses were prepared in *E. coli* DH10Bac bacteria (Invitrogen, San Diego, CA, USA) containing the mini Tn7-replicon. The bacmids generated were isolated following the procedure of the PureLink HiPure Plasmid Miniprep DNA Kit (Life Technologies, Carlsbad, CA, USA) and checked by PCR to confirm the correct gene transposition. Two PCR reactions designed with specific primers to detect either the whole transposition cassette (PCR 1) or the expression cassette (PCR 2) were performed, and an empty bacmid without gene transposition was used as a negative control. Expected size bands were obtained in both PCRs. Then, the transfection of the bacmids in the regulatory Sf9-RVN Glycobac cells (SIGMA-ALDRICH, St. Louis, MO, USA) was performed using 500 ng of bacmids and the protocol of the JetOPTIMUS transfection reagent (PolyPlus) in a monolayer with 0.8 × 10^6^ sf9-RVN cells in ESF-AF media. At 96 hpi the supernatant was harvested, obtaining passage 0 of the baculovirus. These viruses were amplified to generate a high-titer stock. For this purpose, vessels with 15 mL of a suspension of 0.7 × 10^6^ Sf9-RVN cells/mL were infected with passage 0, and 72 h post-infection (hpi), the culture was harvested by centrifugation to obtain the supernatant containing passage 1 and the pellets to be analyzed for protein expression. The baculovirus obtained was titrated by a standard plaque protocol in Sf21 cells (Invitrogen, San Diego, CA, USA). The virus titer was determined as plaque-forming units (PFU).

The rBV passages were also analyzed by PCR using the same two sets of primers for PCR 1 and PCR 2 as were used with the bacmids and the bacmids DNA, as a positive control of PCR amplification, confirming the correct size of the cloned fragments. Amplified fragments were sequenced, confirming that no changes in the cloned genes were introduced during the baculovirus generation processes.

### 2.3. Protein Expression

Once the recombinant baculoviruses (rBVs) were generated expressing each VP2 version, they were tested in insect cells. The rBV passages were analyzed for protein expression in sf-9 RVN cells. To perform this analysis, the pellets from passage 1-infected cell cultures were lysed with RIPA buffer and the lysates were loaded into SDS-PAGE for Coomassie blue stain and Western blot analysis. Western blot was performed using 6xHis monoclonal antibody (TaKaRa, San Jose, CA, USA).

Similar experiments were carried out to determine the best condition for recombinant protein expression in *Trichoplusia ni* pupae as living biofactories [55,56,57]. Different rBV doses between 5000 and 50,000 PFU/insect, insect incubation temperature from 23 to 28 °C, and infection times from 3 to 6 days were tested. Pupae infected under each condition (5 g of pupae biomass) were mechanically homogenized using as extraction buffer 80 mL of Tris 20 mM, NaCl 300 mM, Arginine 50 mM, Tween 80 0.5% pH 7.5. Then, 1 mL of pupae extract was taken from each group and centrifuged at 13,000× *g* for 15 min at 4 °C to split samples into soluble and insoluble fractions. Protein expression and solubility were checked using SDS-PAGE and Coomassie blue staining. Bands corresponding to the recombinant proteins were submitted to densitometry, and relative quantification was carried out with ImageLab 6.1 software analysis (Bio-Rad, Hercules, CA, USA).

### 2.4. Protein Purification

Once pupae batches were generated using the best expression condition for each version, purification of the two recombinant VP2 proteins was performed using the His tag in the C-terminal present in both proteins. Briefly, protein extraction was performed in optimized extraction buffer, Tris 20 mM, NaCl 300 mM, Arginine 50 mM, Imidazole 50 mM, Tween 0.05% pH 7.5, using a colloidal mill. Insect biomasses (62.5 g of pupae) were homogenized in 1 L of extraction buffer, followed by high-pressure homogenization with 1 cycle at 300 bars. Centrifugation removed insoluble fraction, and soluble extract was clarified using depth filtration. IMAC using Histrap Crude FF (Cytiva, Wilmington, DE, USA) resin was performed, and protein elution was achieved using 500 mM of Imidazole concentration. SDS-PAGE analysis helped to select protein fractions that were later dialyzed in PBS 1X pH 7.4.

### 2.5. Protein Characterization

Protein concentrations were measured using SDS-PAGE and band densitometry using BSA curve as reference. Purity of proteins was measured by SDS-PAGE and Coomassie blue staining using band densitometry of gels. Gels were analyzed using ImageLab software from Bio-Rad.

Protein trimerization, necessary for inducing neutralization antibodies, was checked by FPLC-SEC using Superose 6 16/300 (Cytiva Wilmington, DE, USA), equilibrated in PBS 1X pH 7.4, previously calibrated with gel filtration HMW calibration kit (Cytiva, Wilmington, DE, USA).

### 2.6. Mouse Immunization and Challenge

Male and female type I interferon receptor defective mice (IFNAR (−/−)) on A129 Sv/Ev background and A129 mice were used throughout. All mice were matched for age (8 weeks). The mice were housed under pathogen-free conditions and allowed to acclimatize to the biosafety level 3 (BSL3) animal facilities at the Animal Health Research Center (CISA-INIA, CSIC), Madrid, before use. Groups of IFNAR(−/−) mice (n = 5) were intramuscularly immunized following a homologous prime–boost regime consisting of two doses of 30 µg per mouse of VP2-wt or VP2-mod in Freund’s Adjuvant, administered four weeks apart. A group of mice (n = 5) was left immunized with Freund’s Adjuvant (control). Animals were subcutaneously challenged with a lethal dose of EHDV-8 (100 PFU) two weeks post-boost. Sera of immunized and control animals were collected at four weeks post-prime (w.p.p.) and two weeks post-boost (w.p.b.) for the analysis of the humoral immune response. After virus challenge, mice were daily examined for survival and clinical signs, and submandibular blood samples were collected at 3, 5, 7, 10, and 14 d.p.i. for the analysis of viremia and RNAemia by plaque assay in Vero cells and RT-qPCR, respectively. Sera of immunized and control animals were collected at 0, 3, and 5 d.p.i. for the analysis of circulating proinflammatory cytokines.

### 2.7. Determination of Viremia and RNAemia

Blood samples were collected from the submandibular plexus of mice with EDTA as an anti-coagulant. For the analysis of viremia by plaque assay, 50 µL of blood were diluted in PBS1X and centrifuged at 3000 rpm for 10 min. Thereafter, supernatant was removed, and pellet was lysed in 450 µL of sterile water for 2 min. Cell lysis was stopped by adding 50 µL of PBS10X. Then, samples were inoculated into 12-well plates containing semi-confluent monolayers of Vero cells. Following incubation for 1 h, an agar overlay (DMEM-10%-FBS-0.4%-Noble Agar, Becton Dickinson, MD, USA) was added, and the plates were incubated for 5 days at 37 °C in 5% CO_2_. Plaques were fixed with 10% formaldehyde and visualized with 2% crystal violet-PBS.

For the analysis of RNAemia by RT-qPCR, RNA was extracted from 50 µL of blood using TRIzol Reagent (Sigma Aldrich, St. Louis, MO, USA) following the protocol established by the manufacturer. RNAemia was analyzed in duplicate by real-time RT-qPCR specific for EHDV segment 9 (encoding for VP6 and NS4). The real-time RT-qPCR specific for EHDV segment 9 was performed using the primers and probe described by Maan et al. [58]. Only Ct values lower than 38 were considered indicative of viremia (positive), and “No Ct” values were considered as a Ct of 45.

### 2.8. Cytokine Levels in Immunized and Non-Immunized Mouse Sera

Sera from the immunized and non-immunized mice were extracted at 0, 3, and 5 d.p.i. Circulating cytokine levels were analyzed using a multiplex fluorescent bead immunoassay for the quantitative detection of mouse cytokines (ProcartaPlexMouse TH1/Th2 Cytokine Panel 11plex, Invitrogen™, Carlsbad, CA, USA). Samples were analyzed with a MAGPIX system (Luminex Corporation, Austin, TX, USA).

### 2.9. Specific Indirect ELISA for Proteins VP2 and VP7

MaxiSorp plates (Nunc) (Thermo Fisher Scientific, New York, NY, USA) were coated with EHDV-8 VP2 (100 ng per well) or EHDV-2 VP7 [50] (100 ng per well) purified baculovirus-expressed proteins in PBS and incubated overnight at 4 °C. Plates were saturated with blocking buffer (PBS-0.05%-Tween 20-5% skim milk). Individual mice or bovine sera diluted in blocking buffer (1:100) were added and incubated for 2 h at 37 °C. After three washes in PBS-0.05% Tween 20, plates were incubated for 1 h at 37 °C with an anti-mouse-IgG-HRP secondary antibody (Bio-Rad, Hercules, CA, USA) (1:2000) or with an anti-bovine-IgG-HRP secondary antibody (Sigma-Aldrich, San Louis, MO, USA) (1:2000) in blocking buffer. Finally, after three washes in PBS-0.05% Tween 20, the reaction was developed with 50 μL of TMB (Thermo Fisher Scientific, Rockville, MD, USA) and stopped by adding 50 μL of 3 N H_2_SO_4_ (Merck, Darmstadt, Germany). Results were expressed as optical densities (ODs) measured at 450 nm.

### 2.10. PRNT_50_

Two-fold dilutions (from 1:5) of heat-inactivated mouse sera (56 °C for 30 min) were incubated with 100 PFU of EHDV-6 or EHDV-8 for 1 h at 37 °C. Then, samples were inoculated into 12-well plates containing semi-confluent monolayers of Vero cells. Following incubation for 1 h, an agar overlay (DMEM-10%-FBS-0.4%-Noble Agar, Becton Dickinson, Sparks Glencoe, MD, USA) was added, and plates were incubated for 5 days at 37 °C in 5% CO_2_. Plaques were fixed with 10% formaldehyde and visualized with 2% crystal violet-PBS. A 50% plaque reduction neutralization test (PRNT_50_) titer was calculated as the reciprocal (log_10_) of the highest dilution of serum that neutralized 50% of the control virus input. The cut-off was 0.69, log_10_ of the reciprocal of the first dilution 1:5. Negative mouse sera, cells in absence of virus, and cells infected with virus in absence of serum were used as internal controls.

### 2.11. Statistical Analysis

GraphPad Prism version 8.0.1 (GraphPad Software, San Diego, CA, USA) was used to analyze data. Each immunized group’s survival curve was compared to that of non-immunized animals using Log-rank test. The mean viremia and RNAemia responses between groups were compared by multiple t-test analysis using the Sidak–Bonferroni method. Differences between groups regarding the PRNT_50_, VP2, or VP7 ELISA assays, and circulating cytokine levels were conducted by two-way ANOVA with a post hoc Tukey test for multiple comparisons. A *p*-value lower than 0.05 was considered significant in all cases.

## 3. Results

### 3.1. Protein Design, Expression, Purification, and Characterization

The VP2 capsid protein of EHDV-8 consists of 970 amino acids and assembles into trimeric structures, forming a complex of approximately 300 kDa [38]. The complete sequence of the VP2 capsid protein from EHDV serotype 8 (GenBank accession no. OP897266.1, derived from the isolate EHDV-8 2022.TE.50459.1.2) was used to generate different gene variants for recombinant expression.

Based on structural information from the homologous protein of BTV, a member of the same viral family, the VP2 monomer can be divided into four domains: (1) a terminal domain (amino acids 121–162 and 850–970), the least exposed region, likely involved in trimer formation; (2) a hairpin domain (amino acids 50–120), potentially mediating interaction with VP5; (3) the body domain (amino acids 163–190 and 408–849); and (4) the immunogenic domain (amino acids 191–407) [38,52].

**Figure 1:**
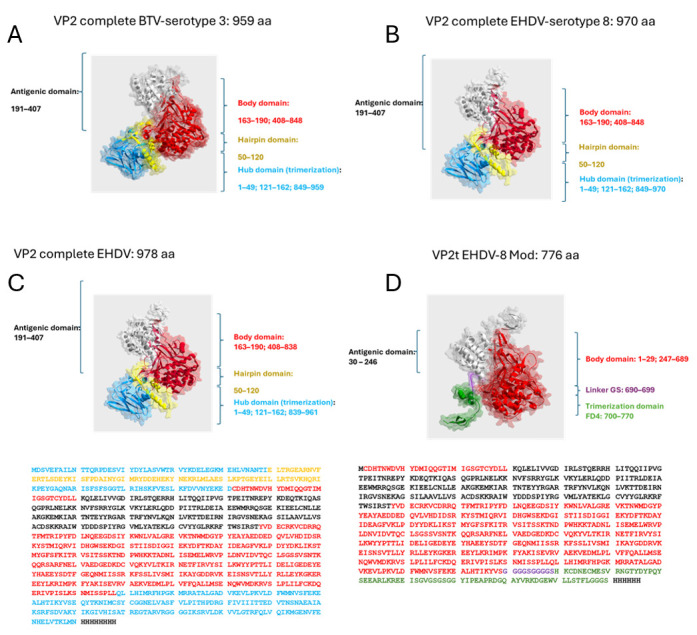
(**A**,**B**) A 3D predicted structure comparison between BTV and EHDV viruses defining the different protein domains (Hub domain, Hairpin domain, Antigenic domain, and Body domain). (**C**,**D**) A 3D predicted structure of the wild-type VP2 protein of EHDV (labeled “Full-length VP2 EHDV-8”) and the fusion protein of the present invention (labeled “VP2t EHDV-8 Mod”). The trimerization domain includes a GGGS linker at the C-terminus followed by a polyhistidine tag.

To enhance recombinant VP2 expression, a truncated construct was designed, lacking the hub (discontinuous trimerization region) and hairpin domains. These were substituted with the T4 fibritin foldon (FD4), a continuous trimerization motif. The engineered protein comprised 776 amino acids, incorporating a flexible VP2–FD4 linker and a C-terminal His-tag to facilitate purification. The deletion of aggregation-prone hydrophobic residues improved solubility, while FD4 restored the trimerization required for neutralizing antibody induction. Structural models of both the native and engineered proteins were generated using Swiss-PdbViewer (Figure 1).

**Figure 2:**
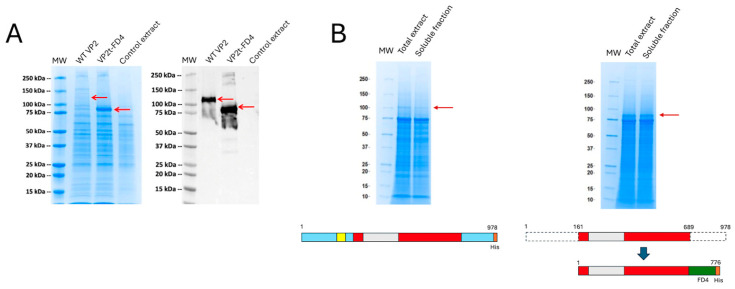
Protein expression SDS-PAGE analysis. (**A**) VP2-EHDV-8 and VP2t-EHDV-8 Mod proteins expression in sf9-RVN insect cells, analyzed by Coomassie blue staining and detected by Western blot using an anti-Histidine monoclonal antibody. Red arrows indicate the recombinant protein. (**B**) Protein expression in infected pupae extracts, total and soluble extracts analyzed by Coomassie blue stain. Red arrows indicate the recombinant protein. Below each gel, there is a schematic representation of the protein domains included in the constructs.

Recombinant baculoviruses expressing either the wild-type or engineered VP2 proteins were used to infect insect cells and pupae. Significant differences in expression were observed, with the engineered protein achieving higher yields (Figure 2A,B). Although the wild-type VP2 showed robust expression, it remained largely insoluble under all tested conditions (Figure 2B). Optimal productivity was achieved with pupae infected at 50,000 PFUs and incubated for six days at 23 °C. In contrast, the engineered VP2 reached maximum expression after five days post-infection and displayed markedly improved solubility, greatly facilitating its purification (Figure 2).

**Figure 3:**
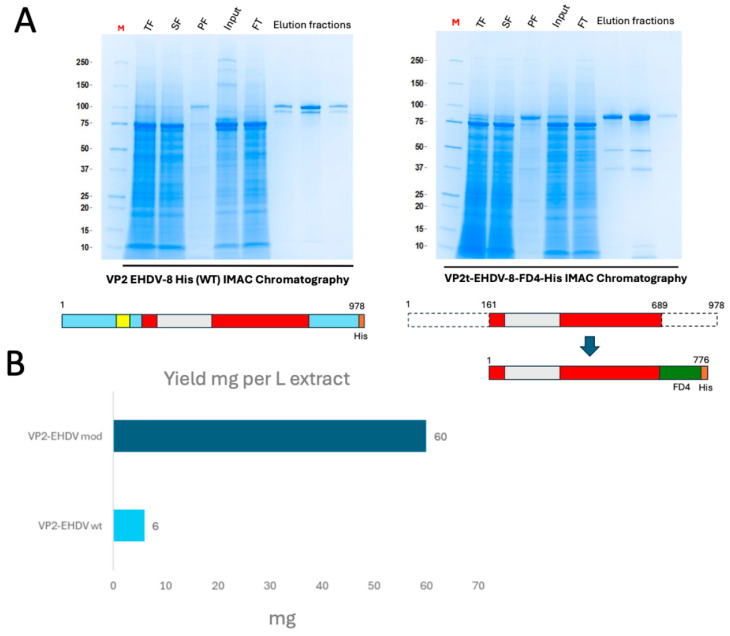
(**A**) IMAC purification of pupae-derived recombinant VP2 proteins analyzed by Coomassie blue staining. (**B**) Calculation of yields of purified full-length and modified protein versions obtained per liter of pupae extract. Proteins were quantified by densitometry of the gels with a BSA curve.

Purification of the pupae-derived wild-type VP2 was performed by immobilized metal affinity chromatography (IMAC) (Figure 3A). Despite optimization of extraction buffers, considerable losses occurred due to low solubility. After conditioning in PBS 1X (pH 7.4), protein purity exceeded 80% in a single-step purification, but final yields were limited to approximately 6 mg per liter of pupae extract (0.1 mg/g pupae) (Figure 3B). The engineered VP2, in contrast, yielded around 60 mg per liter of pupae extract—a tenfold increase compared to the wild-type—with a final purity above 85% (Figure 3B).

**Figure 4:**
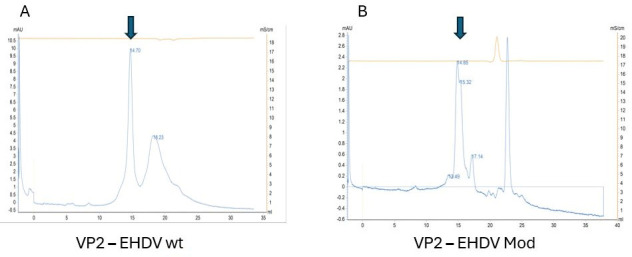
FPLC-SEC analysis supporting the presence of trimeric structures in both VP2 molecules expressed. (**A**) corresponds to the wild-type VP2 protein and (**B**) to the modified version. Trimers are indicated as expected trimer elution according to column calibration. Arrows correspond to trimers.

Protein VP2 assembles into triskelion-shaped trimers, whose conformation seems to be essential for the processes of viral assembly and infectivity and plays a significant role in modulating the accessibility and stability of neutralizing epitopes [38,51,52]. To evaluate protein conformation, trimerization was analyzed by size-exclusion chromatography. The retention times obtained in FPLC-SEC with both wild-type and engineered VP2 proteins suggest a trimeric organization, as interpolation on the calibration curve indicates an apparent molecular mass of approximately 250 kDa (Figure 4). Nevertheless, unambiguous characterization of the protein’s quaternary structure will require complementary techniques, such as Cryo-EM or HPLC-SEC-MALS.

### 3.2. Recombinant VP2 Proteins Induced Neutralizing Antibodies and Protected Against a Virulent EHDV Challenge

**Figure 5:**
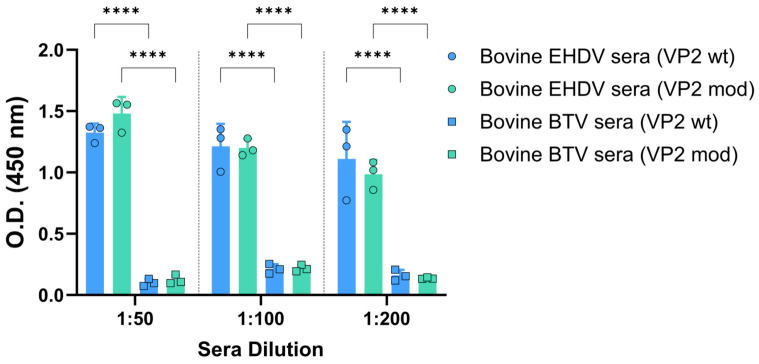
Recognition of the pupae-derived VP2 proteins by sera from naturally infected bovines with EHDV or BTV. Bovine sera were analyzed by indirect ELISA for the presence of IgG immunoglobulins against wild-type or engineered VP2 of EHDV-8. The results were expressed as optical densities (O.D.) measured at 450 nm. Bars represent individual values for each animal. The sera dilutions were 1:100. Bars represent the mean values of each group, points represent individual values, and error bars represent SD. Asterisks denote significant differences between groups. No statistical differences were found between O.D. values corresponding to antibody responses of EHDV sera against wild-type or engineered VP2. **** *p* value < 0.0001, using two-way ANOVA (post hoc Tukey test for multiple comparisons).

First, we assessed the antigenicity of the recombinant VP2 proteins by indirect ELISA using sera from bovines naturally infected with either EHDV or BTV. As shown in Figure 5, only sera from animals infected with EHDV recognized the two recombinant EHDV-derived proteins, whereas sera from BTV-infected bovines showed no reactivity, which indicates the absence of cross-reactivity between VP2 proteins from distinct orbiviral origins. In addition, no differences were found between the IgG responses of sera from EHDV-infected bovines against the wild-type and the modified VP2 proteins (Figure 5).

Recently, IFNAR(−/−) mice have been established as a suitable animal model for EHDV infection and vaccine evaluation [59]. To test the immunogenicity and protective capacity of the two recombinant VP2 proteins, three groups of IFNAR(−/−) mice (n = 5 per group) were subjected to a prime–boost vaccination regimen. Two groups received intramuscular injections of 30 μL vaccine containing 30 μg of either wild-type or engineered VP2 protein, formulated with Freund’s Adjuvant (complete for the prime and incomplete for the boost). A third group served as a negative control and was administered adjuvant only. The booster immunization was delivered four weeks after the prime. Two weeks after the booster dose, mice were subcutaneously challenged with a lethal dose (100 PFU) of EHDV-8. Subsequently, the mice were monitored for survival, and viremia and RNAemia were analyzed by plaque assay and RT-qPCR, respectively.

**Figure 6:**
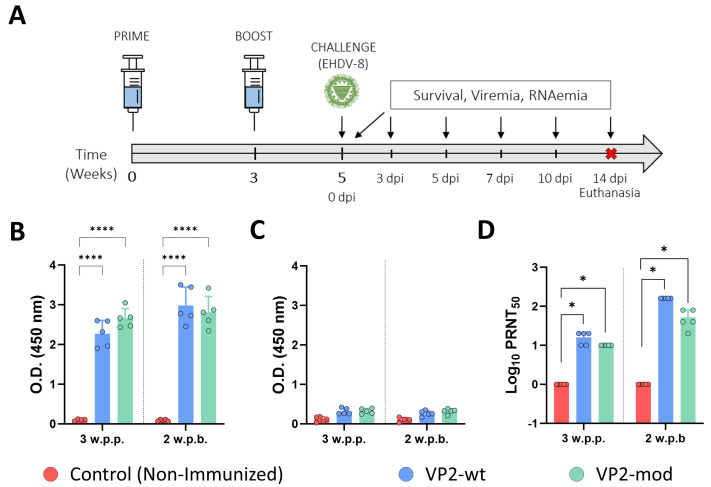
The induction of a humoral immune response specific to EHDV in IFNAR(−/−) immunized mice. (**A**) After the prime–boost immunization of groups of IFNAR(−/−) mice (n = 5) with wild-type or engineered VP2 proteins, the animals were challenged with EHDV-8. A group served as a control. (**B**,**C**) Induction of specific VP2 (**B**) or VP7 (**C**) IgG by indirect ELISA in vaccinated animals. Sera dilutions were 1:100. (**D**) PRNT_50_ against EHDV-8 to determine neutralizing antibody titers in immunized animals. Serum samples were collected at 3 w.p.p. and 2 w.p.b. to measure antibody responses by ELISA and nAbs titers against EHDV-8. Bars represent the mean values of each group, points indicate the individual value of each animal, and error bars represent SD. Asterisks denote significant differences between groups. * *p* value < 0.05, **** *p* value < 0.0001, using two-way ANOVA (post hoc Tukey test for multiple comparisons). The red cross means sacrifice mice.

We evaluated the induction of a humoral immune response by the VP2-based subunit vaccine candidates. To do so, we collected sera at 4 weeks post-prime-vaccination (w.p.p.) and 2 weeks post-boost-vaccination (w.p.b.), and assessed the presence of IgG antibodies specific to protein VP2 of EHDV-8. Antibodies specific to the VP2 protein of EHDV-8 could be detected in all mice immunized with either wild-type or engineered VP2 proteins (Figure 6B). Levels of VP2-IgG slightly increased after the booster in both immunization groups (Figure 6B). We also performed an indirect ELISA test for the detection of IgG antibodies against the recombinant protein VP7 of EHDV. No antibodies specific to protein VP7 could be detected prior to challenge in any of the immunization groups (Figure 6C), which highlights the DIVA character of both subunit vaccine candidates. In addition, we measured neutralizing activity against EHDV-8 or EHDV-6 in sera collected by classic PRNT_50_. Immunized animals displayed detectable titers of homologous virus-NAbs after one dose and high PRNT_50_ titers after two doses of each recombinant protein (Figure 6D). No statistical differences were found between the immunization groups regarding EHDV-8 NAbs titers. Unsurprisingly, neither the wild-type VP2 nor the modified VP2 vaccine candidates induced cross-neutralizing responses against EHDV-6. Overall, immunization with either VP2-wt or VP2-mod leads to the induction of identical humoral immune responses against EHDV, indicating that the modifications introduced in the sequence of protein VP2 do not alter its immunogenicity.

**Figure 7:**
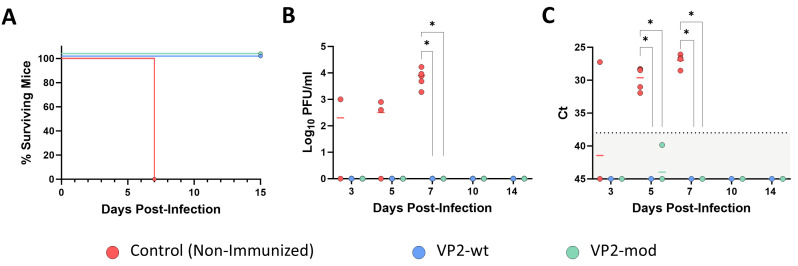
Protection of immunized IFNAR(−/−) mice against lethal challenge with EHDV-8. (**A**) Survival curves after challenge with EHDV-8. Survival curves of immunized mice were significantly different from the control group (by Log-rank test, *p* value < 0.05). (**B**) Infectious virus titers in blood of IFNAR(−/−) mice after challenge. Points represent individual log_10_ PFU/mL values for each mouse, and lines of corresponding color represent mean log_10_ PFU/mL value of each group. Mann–Whitney U test was used to determine statistical differences between groups (* *p*-value < 0.05). (**C**) Viral RN levels in blood of immunized and non-immunized IFNAR(−/−) mice, analyzed by RT-qPCR after challenge. Expression of mRNA of segment 9 was quantified at 3, 5, 7, 10, and 14 d.p.i., and results were expressed as Ct (left y axis). The RT-qPCR specific to EHDV segment 9 was performed as described by Mann et al. [58]. Individual Ct values for each animal are represented by points, and lines of the corresponding color indicate the mean Ct value of each group. A Ct value of 45 was considered for “No Ct” values. A Mann–Whitney U test was used to determine statistical differences between groups (* *p*-value < 0.05). The dashed line is the negative limit of the assay.

After challenge with a lethal dose of EHDV-8, control mice showed clinical signs characteristic of EHDV infection in IFNAR(−/−) mice from 4 d.p.i. At day 7 post-infection, all control animals succumbed to EHDV infection (Figure 7A). In contrast, immunization with either wild-type or engineered VP2 proteins protected animals against lethal challenge with EHDV-8, leading to 100% survival rates after challenge (Figure 7A). Control animals also displayed high levels of viremia and RNAemia at 5 and 7 days post-infection (d.p.i.) (Figure 7B,C). In contrast, no infectious virus or viral RNA could be detected in the blood of animals immunized with either VP2-wt or VP2-mod throughout the experiment (Figure 7B,C). Altogether, these data confirm that both protein versions confer robust protection against EHDV infection. More importantly, no differences exist between VP2-wt and VP2-mod in terms of protection efficacy.

**Figure 8:**
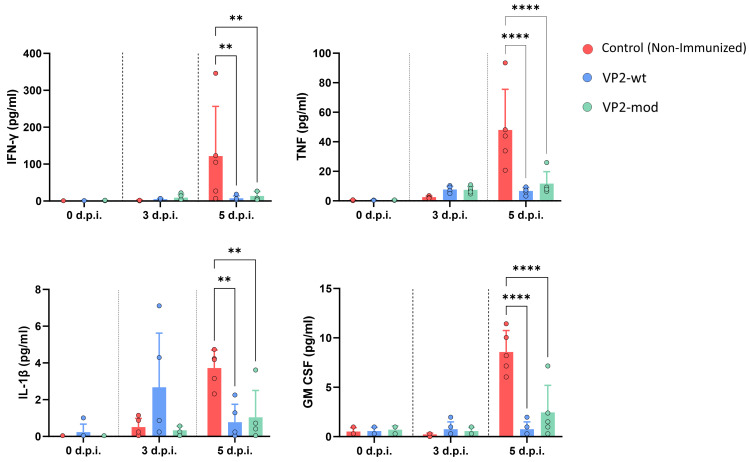
Determination of circulant cytokine levels in sera from immunized and non-immunized animals at different time points post-infection. Individual values of each mouse are represented by points, the mean values of each group by bars and error bars represent SD. Asterisks indicate significant differences between groups. ** *p* value < 0.0021, **** *p* value < 0.0001, using two-way ANOVA (post hoc Tukey test for multiple comparisons).

Vaccination against EHDV can prevent the induction of the cytokine storm, a characteristic pathogenic feature caused by EHDV infection in natural hosts and IFNAR(−/−) mice [59,60,61]. Therefore, we tested whether our VP2-based subunit vaccine candidates also blocked the induction of the cytokine storm by measuring circulating levels of IFN-γ, TNF, IL-1β, and GM-CSF in control and immunized IFNAR(−/−) mouse sera after challenge with EHDV-8. After challenge, control animals showed a step rise in the circulating levels of these proinflammatory cytokines at day 5 post-infection (Figure 8). Animals from both VP2-wt and VP2-mod immunization groups displayed significantly lower levels of IFN-γ, TNF, IL-1β, and GM-CSF compared to the control animals. Interestingly, some VP2-wt immunized mice exhibited elevated levels of IL-1β at 5 d.p.i. (Figure 8). Again, no differences were found between both vaccine candidates at any time point evaluated, which indicates that both vaccine candidates efficiently block EHDV-induced hypercytokinemia.

## 4. Discussion

Since the first detection of EHDV-8 in 2022, no other serotype of EHDV has been detected in Europe, although EHDV-6 currently circulates in North Africa [10]. EHDV-8 has become a major problem for the primary sector, as it has significantly affected the cattle population but also wildlife in Spain, France, Portugal, and Italy [14,19]. Lately, between April 2024 and February 2025, 182 cases were reported in Spain, 46 in Portugal, and 18 in France (source: Spanish Ministry of Agriculture). Climate change and the involvement of wildlife reservoirs make eradication particularly difficult. In consequence, systematic vaccination is the only effective strategy to reduce the incidence of the virus in livestock populations, as was the case with BTV [62]. To date, two vaccines have been approved by the European Medicines Agency: an inactivated whole-virus vaccine (Hepizovac; Vetia Animal Health, Madrid, Spain) and a subunit DIVA vaccine based on the VP2 protein expressed in insect cells (Syvac EH Marker; Syva, León, Spain). Here, we engineer the VP2 protein of EHDV for superior expression and full protective immunity in a subunit vaccine.

The VP2 protein of EHDV forms the outer capsid layer of the virion and is essential for viral assembly and host cell entry [36,37,38]. Owing to its antigenic properties, VP2 represents a major target for vaccine development [63,64,65]. However, the recombinant production of VP2 is limited by low stability and poor yields, representing a significant barrier for the generation of safe and effective subunit vaccines. This conclusion is based on the results presented in this study. Previous work in which the VP2 protein was produced from EHDV-2 and EHDV-6 [46] or from EHDV-8 [47] did not report the yield of the expression and purification processes. These studies also did not provide Coomassie blue–stained SDS-PAGE gels or protein stability assays. This lack of comparable data makes it difficult to evaluate the expression of our modified VP2 protein relative to other VP2 proteins previously expressed using the baculovirus system.

To overcome these limitations, the VP2 protein from EHDV serotype 8 was engineered with targeted modifications at both the amino and carboxyl termini of its coding sequence. For the rational design of the VP2 molecule from EHDV, the structural information of the homologous protein from BTV was used as a reference, since no structural data are available for EHDV. According to Zhang et al., BTV VP2 contains four distinct domains, as previously described, each of which may play a specific role [52]. The hub domain is proposed to form the core required for trimerization. The hairpin domain is involved in the interaction with the other outer capsid protein, VP5. The body domain provides a scaffold that supports the antigenic domain.

Our objective was to reduce the molecular weight of VP2, since numerous studies have shown that size reduction enhances solubility. For instance, in *E. coli*, the yield of soluble proteins decreases dramatically once the molecular weight exceeds ~60 kDa [66]. Importantly, this strategy aimed to avoid disrupting the structural integrity of the antigenic domain.

Recombinant expression was performed using a baculovirus-mediated system in *Trichoplusia ni* pupae (CrisBio^®^ technology, Algenex, Tres Cantos, Spain), employed as living biofactories. The engineering of VP2 resulted in up to a tenfold increase in protein yields compared with the wild-type sequence, while maintaining the trimeric conformation of the recombinant protein. Once the structural integrity of the recombinant proteins was assessed, both wild-type and engineered VP2 variants were formulated and used to immunize IFNAR(−/−) mice, a model susceptible to EHDV infection [59]. Immunogenicity was evaluated by measuring virus-neutralizing antibody responses, and protective efficacy was assessed through lethal challenge with a virulent EHDV-8 strain. Both engineered and wild-type VP2 formulations elicited comparable neutralizing antibody responses in IFNAR(−/−) mice. Furthermore, immunization with either formulation conferred full protection against lethal EHDV-8 challenge. We could not observe clinical signs of disease or viral replication in either immunization group after challenge with EDHV-8. In ruminant hosts and IFNAR(−/−) mice, EHDV infection is related to the induction of an exacerbated proinflammatory immune response that may contribute to the vascular injury caused by virus replication in the endothelium [59,60,61]. Importantly, immunization with the engineered or wild-type VP2 proteins prevented animals from developing this EHDV-8-induced cytokine storm, which may reflect the abrogation of the virus pathogenesis by both vaccine candidates. Therefore, it is demonstrated that the rational engineering of the VP2 protein of EHDV-8 significantly improved recombinant expression yields without compromising structural integrity or immunogenicity. These findings also demonstrate the feasibility of producing high-quality VP2 antigens in *T. ni* pupae using CrisBio^®^ technology and support their potential application in the development of subunit vaccines against EHDV.

VP2 structural information was not available for the rational design of a new protein. However, nowadays, thanks to new AI technologies such as AlphaFold2, the lack of tertiary and quaternary protein structures is no longer a limitation. The prediction of 3D structures has enabled the rational design of novel molecules for which no experimental data previously existed. In order to study the structure of VP2, the 3D structures of VP2 monomers from both BTV (serotype 3) and EHDV were generated from their complete amino acid sequences using AlphaFold 2. The dissection of the structural domains of complete VP2 performed by Zhang et al. [52], in combination with 3D visualization, enabled the identification of cleavage points in the monomer at regions displaying unfolded secondary structure, while also allowing the deletion of entire domains, such as the hub and hairpin domains. Without these predictions, the identification of these cleavage sites would not have been straightforward, and the generation of multiple constructs with different gene sequences would have increased exponentially, slowing the possibility of responding with new structures to emergent diseases such as EHD. Therefore, the hub and hairpin domains were removed. Sequence analysis reveals that 47% of hydrophobic amino acids present in the hub and hairpin domains. This may explain the increase in solubility and expression of the mutated version of the protein with respect to the original sequence.

Preliminary studies suggested that VP2 trimerization is required for the generation of neutralizing antibodies, and to preserve this property, we introduced the trimerization domain from bacteriophage T4, known as Foldon (FD4). In this way, we obtained a protein of 770 amino acids, compared to the original 956-residue molecule. The application of AI in protein design may allow the creation of modified proteins with specific functions and structures that perform desired tasks. This is a breakthrough in biotechnology and, particularly, in the development of subunit vaccines based on recombinant proteins; these are frequently difficult to produce, limiting the success in creating cost-efficient, safe, and effective vaccines [67,68]. In this sense, the modified version of the EHDV-8 VP2 antigen represents a successful example that illustrates the capacity of AI to assist researchers in rational immunogen design, with significant impact on vaccine development.

Aside from the antigenicity of the recombinant vaccine candidate based on a modified protein VP2 of EHDV-8, a highly significant aspect of this work is the generation of a DIVA-compatible vaccine candidate through a more efficient production process, yielding a stable antigen able to induce protective immunity comparable to that of currently licensed vaccines. The high-yield production platform has the potential to ensure a more efficient supply for large-scale vaccination campaigns, while substantially lowering manufacturing costs, which alternative VP2 constructs or production systems (CrisBio^®^, Algenex, Tres Cantos, Spain, versus insect cell expression) cannot achieve. Cost-effective production is critical for making vaccines commercially viable and for providing tangible benefits to livestock farming.

Immunocompromised IFNAR(−/−) mice have been widely used to evaluate the efficacy of vaccines against a wide range of viral pathogens, including orbiviruses such as BTV and African Horse Sickness virus [69,70]. The establishment of IFNAR(−/−) mice as a valid infection model for BTV and AHSV enabled conducting preclinical trials of novel vaccine candidates as a preliminary step, prior to evaluation in natural hosts [54]. Importantly, vaccine-induced adaptive immune responses against BTV and AHSV do not significantly differ between IFNAR(−/−) mice and natural hosts [54,71]. Although IFNAR(−/−) mice have been recently established as a valid model for EHDV study and preclinical trials of recombinant vaccines against EHDV have been conducted [50,59,72], extrapolation to ruminant species is uncertain. In this regard, vaccination trials in cattle are currently underway to validate the observations obtained in the IFNAR(−/−) mouse model. Preliminary results suggest that the immunization of calves with the modified VP2 protein efficiently induces neutralizing antibody responses, at levels comparable to those achieved with a complete inactivated virus.

## 5. Conclusions

In summary, this study presents a promising recombinant vaccine candidate against EHDV-8 based on a high-yield modified version of protein VP2. This immunogenic version of protein VP2 could influence present and future subunit vaccine design against EHDV and may promote the implementation of cost-effective vaccine approaches against this viral disease. Furthermore, the VP2 engineering strategy applied in the case of EHDV could be extended to enhance the production of capsid proteins from other orbiviruses within the same family, such as BTV or AHSV.

## 6. Patents

The European patent application EP24383330.8, entitled *Sedoreoviridae* vaccines based on truncated VP2 capsid proteins, was submitted on 9 December 2024.

## Data Availability

All data created in this study have already been shown in the manuscript. No more new data is available.
